# From childhood physical and emotional abuse to self-harm: the mediating effect of sleep disorders among a sample of male prisoners

**DOI:** 10.1192/j.eurpsy.2025.2327

**Published:** 2025-08-26

**Authors:** I. Mlouki, A. Ben Haouala, E. Hariz, A. Silini, Y. Abess, M. Boussaid, F. Zaafrane, S. El Mhamdi

**Affiliations:** 1 Preventive and Community Medicine, University Hospital Tahar Sfar, Mahdia; 2 Research Laboratory “Vulnerability to psychotic disorders”, University of Monastir, Monastir; 3 Forensic Medicine, University Hospital Tahar Sfar, Mahdia, Tunisia

## Abstract

**Introduction:**

According to literature, the link between childhood trauma and mental health disorders is well-established. However, focusing on the mechanisms explaining this pathway remains insufficiently studied mainly among incarcerated population.

**Objectives:**

The current study aimed to assess the mediation effect of sleep disorders in the path from childhood physical neglect and emotional abuse to suicide and self harm behaviors among male prisoners.

**Methods:**

We conducted a cross sectional study among incarcerated males in Mahdia prison on April 2023. The participation was anonymous, voluntary and the measurement tool was self-administrated. Illiterate participants were interviewed by investigator doctors. We assessed physical and emotional abuse using items from the validated Arabic version of the World Health Organization ACE questionnaire. Self-directed violent behaviors were subdivided into suicidal behaviours and self harm. The Hospital Anxiety and Depression Scale and The Pittsburgh sleep quality index were also used.

**Results:**

A total of 540 prisoners were recruited with a response rate of 74.6%. Their mean age was 33.75± 10.89 years. Among participants, 73% reported emotional abuse and 39.2% had experienced physical neglect during their childhood. In the six previous months, 35% of them had suicidal thoughts and 45.9% were engaged in self-harm. Anxiety and depression were screened among 83.2% and 79.6% of them respectively. During incarceration, 88.4% of prisoners reported sleep disorders.

After adjusting for anxiety and depression, we found that physical neglect and emotional abuse predict self harm behaviors through sleep disorders among male prisoners (%mediated= 28.5% and 15% respectively, p< 0.001).

**Conclusions:**

Our study emphasize the need for preventing and screening childhood trauma and implementing mental health approaches in jail in order to deal with sleep disorders and to prevent suicidal behaviors among prisoners.

**Disclosure of Interest:**

None Declared

